# The Association Between Illicit Drug Use and the Duration of Renal Replacement Therapy in Patients With Acute Kidney Injury From Severe Rhabdomyolysis

**DOI:** 10.3389/fmed.2020.588114

**Published:** 2020-11-09

**Authors:** Andy K. H. Lim, Meor Azraai, Jeanette H. Pham, Wenye F. Looi, Caitriona Bennett

**Affiliations:** ^1^Department of General Medicine, Monash Health, Clayton, VIC, Australia; ^2^Department of Nephrology, Monash Health, Clayton, VIC, Australia; ^3^Department of Medicine, School of Clinical Sciences, Monash University, Clayton, VIC, Australia

**Keywords:** rhabdomyolysis, acute kidney injury, dialysis, renal replacement therapy, illicit drug use

## Abstract

**Background and Aims:** Acute kidney injury is a known complication of severe rhabdomyolysis. In patients who present to hospital with rhabdomyolysis, illicit drug use is associated with a higher risk of acute kidney injury needing renal replacement therapy (RRT), independent of the peak serum creatine kinase level. The aim of this study was to assess if RRT duration and renal outcomes were also worse in illicit drug use-associated rhabdomyolysis.

**Methods:** We conducted a cohort study of adult patients who presented to Monash Health (Jan 2011–June 2020) with rhabdomyolysis and required RRT. Patients with isolated myocardial injury and cardiac arrest were excluded. We used survival analysis to examine the time to RRT independence, utilizing the Fine-Gray competing risks regression and death as the competing event. A subdistribution hazard ratio (SHR) < 1.0 represents a relatively greater duration of RRT and a worse outcome.

**Results:** We included 101 patients with a mean age of 58 years, of which 17% were cases associated with illicit drug use. The median peak creatine kinase level was 5,473 U/L (interquartile range, 1,795–17,051 U/L). Most patients (79%) initiated RRT within 72 h of admission, at a median serum creatinine of 537 μmol/L (interquartile range, 332–749 μmol/L). In the competing risks analysis, the estimated SHR was 1.48 (95% CI: 0.78–2.84, *P* = 0.23) for illicit drug use, 0.87 (95% CI: 0.76–0.99, *P* = 0.041) for the log-transformed peak creatine kinase, and 0.41 (95% CI: 0.25–0.67, *P* < 0.001) for sepsis. A 50% cumulative incidence of RRT independence occurred at 11 days (95% CI: 8–16 days). Only 5% of patients remained on RRT at 3 months.

**Conclusion:** In rhabdomyolysis-associated acute kidney injury, it is unlikely that patients with illicit drug use-associated rhabdomyolysis require a longer duration of RRT compared to patients with rhabdomyolysis from other causes.

## Introduction

Rhabdomyolysis is a clinical syndrome of skeletal muscle breakdown. During the process of muscle injury, the cellular contents leak into the systemic circulation, resulting in severe alterations in the composition of body fluids and electrolytes. The development of acute kidney injury (AKI) is among the most serious complications, which is driven by myoglobin nephrotoxicity, oxidative stress, and inflammation. The severity of rhabdomyolysis varies depending on the cause and the duration of the initial insult and can be estimated by using the serum creatine kinase (CK) level as a surrogate marker.

Rhabdomyolysis is due to many causes which are broadly categorized as inherited or acquired. Inherited causes include genetic defects responsible for several metabolic myopathies. Acquired causes are more common, and most are attributed to drugs and toxins, infections, trauma, or exertional injury. In young adults, illicit drugs and intoxications are increasingly prevalent as a cause of rhabdomyolysis. These illicit drugs include heroin, cocaine, amphetamines or methamphetamines (“ice” or “meth”), cannabinoids (tetrahydrocannabinol, and its synthetic variants like “spice”), ecstasy (MDMA), all of which have been reported to be associated with rhabdomyolysis (1–4).

We previously showed that around 50% of patients admitted to our hospital network with rhabdomyolysis experienced AKI, and around 5% required renal replacement therapy (RRT) (1). We also demonstrated that patients with rhabdomyolysis associated with illicit drugs had a higher odds of AKI and needing RRT, independent of the peak serum CK level. We postulated that illicit drugs could be directly nephrotoxic. However, there are limited publications regarding the determinants of RRT duration once initiated. It is unclear if illicit drug users require a longer duration of RRT compared to patients with rhabdomyolysis from other causes, or more frequently become dialysis dependent. Understanding the duration of RRT may assist in logistical arrangements (site transfers), resource allocation (planning for ongoing outpatient RRT), and providing the patient with an expected recovery timeframe (rehabilitation planning).

In this study, we aimed to determine the factors which influence the duration of RRT in patients with rhabdomyolysis and AKI, and to specifically test the hypothesis that patients with rhabdomyolysis associated with illicit drug have a worse RRT outcome than patients who did not use illicit drugs.

## Methods

### Study Design and Setting

We conducted a retrospective, cohort study of adult patients (≥18 years) admitted to Monash Health acute hospitals where RRT is supported (Monash Medical Centre, Dandenong Hospital) between 1-Jan-2011 and 30-June-2020. Monash Health is a large hospital network in the state of Victoria, Australia. The network is located in the south-east region of Melbourne, and we provide healthcare services to approximately one quarter of the population of Melbourne. This study was approved by the Monash Health Human Research Ethics committee as a quality initiative and individual patient consent was not required (RES-20-0000-148Q).

### Participants

We used the ICD-10 codes to identify patients with both muscle injury and RRT. From the search results, we systematically reviewed the electronic medical records and biochemistry results. Patients were eligible for inclusion if they had clinical and biochemical evidence of rhabdomyolysis, and received acute RRT for AKI. Patients receiving long-term dialysis in any form prior to admission were not eligible. From the eligible patients, we excluded those with (1) a CK rise due to isolated myocardial injury such as myocardial infarction, myocarditis, or cardiac arrest, (2) emergency cardiovascular surgery (e.g., abdominal aortic aneurysm rupture, coronary artery bypass surgery), (3) primarily toxicological indication for RRT (e.g., lithium overdose), and (4) duplicated entries arising from between hospital transfers within our network. All patients had bladder catheterization and sonographic exclusion of urinary tract obstruction as part of the assessment of AKI. Other studies such as screening for glomerulonephritis (including vasculitis and autoimmune diseases) and kidney biopsy were performed at clinician discretion. Patients were followed from the initiation of RRT until cessation of RRT, death or 90 days if they remained on RRT.

### Primary Outcome and Renal Replacement Therapy Information

The primary outcome of the study was the duration of RRT, represented by the time to RRT independence. For survival analysis purposes, the time at risk begins on the day of RRT initiation and ends on the last day of RRT or death (as a competing risk). Patients were censored at 90 days after initiation of RRT as these patients were considered to have non-recovery (dialysis-dependent) by this time, as per the Kidney Disease Improving Global Outcomes (KDIGO) recommended timelines for defining CKD after an episode of AKI (5). Initiation, cessation, and RRT prescription were performed at the discretion of the attending intensivist or nephrologist. For continuous veno-venous hemofiltration (CVVH) or hemodiafiltration (CVVHDF), we used the Baxter Prismaflex AN69 ST100 set (surface area 1 m^2^). For sustained (slow) low-efficiency dialysis (SLED), we used the Fresenius AV600S polysulfone filter (surface area 1.4 m^2^). For intermittent hemodialysis (IHD), we used the Fresenius FX80 high flux polysulfone filter (surface area 1.8 m^2^) with a maintenance regimen of alternate daily 4-h sessions (on average).

### Variable Definitions

We defined the biochemical evidence of rhabdomyolysis as a serum CK level greater than five times normal (>1,000 U/L). The potential causes of rhabdomyolysis were grouped into non-mutually exclusive categories, recognizing that some causes were multi-factorial. We determined illicit drug use status from the clinical history and toxicology results, and grouped them into non-mutually exclusive categories, as polysubstance abuse was common. We defined chronic kidney disease (CKD) staging according to the baseline estimated glomerular filtration rate (eGFR) routinely reported by Australian laboratories using the CKD-EPI equation, using the strategy described by Siew et al. (6) to determine baseline kidney function. For clinical purposes, CKD was defined as an eGFR < 60 ml/min/1.73 m^2^ and advanced CKD was defined as an eGFR < 30 ml/min/1.73 m^2^. The presence of sepsis was determined using the Sepsis-3 criteria (7). As central venous pressure measurements and baseline weights were not always available, we used the clinical assessment of the treating team to define fluid overload (jugular or central venous pressure, peripheral edema, pulmonary congestion), supplemented by information on ultrafiltration goals from the RRT prescription at commencement. The major comorbidities were coded as binary variables except for CKD staging. The potential nephrotoxins examined were intravenous iodinated contrast used for imaging studies and certain antibiotics (vancomycin, aminoglycosides), which were recorded as binary variables of exposure.

### Statistical Analysis

For continuous data with normal distributions, we report the mean and standard deviation (SD). For highly skewed data, we report the median and interquartile range (IQR). We used Student's *t* test to compare group means for normally distributed data, and the Wilcoxon rank-sum (Mann-Whitney) test to compare equality of distributions for significantly skewed data. The association between categorical variables were tested using a chi-squared (χ^2^) analysis or Fisher's exact test where low cell counts were encountered. We used the Fine-Gray (subdistribution hazard) survival analysis model for competing risk to analyze the time-to-event outcome and report the subdistribution hazard ratio (SHR) and cumulative incidence function (8). From the univariable analysis, we included the main epidemiological factor of illicit drug use status and any potential confounders for inclusion in the multivariable model. In the final multivariable model, we retained covariates with a *P* < 0.05 or covariates which changed the SHR estimates for illicit drug use status by more than 10% if dropped from the model. The proportional subdistribution hazards assumption was tested by introducing time-varying coefficients and determining if time interactions were statistically significant. For additional rigor, we also analyzed the effect of covariates on cause-specific hazards using Cox regression and present the hazard ratio (HR) for side-by-side comparison with the Fine-Gray model. All analysis was performed using STATA version 16 (StataCorp, Texas USA). A *P* < 0.05 was considered statistically significant.

## Results

### Patient Characteristics

**Figure 1:**
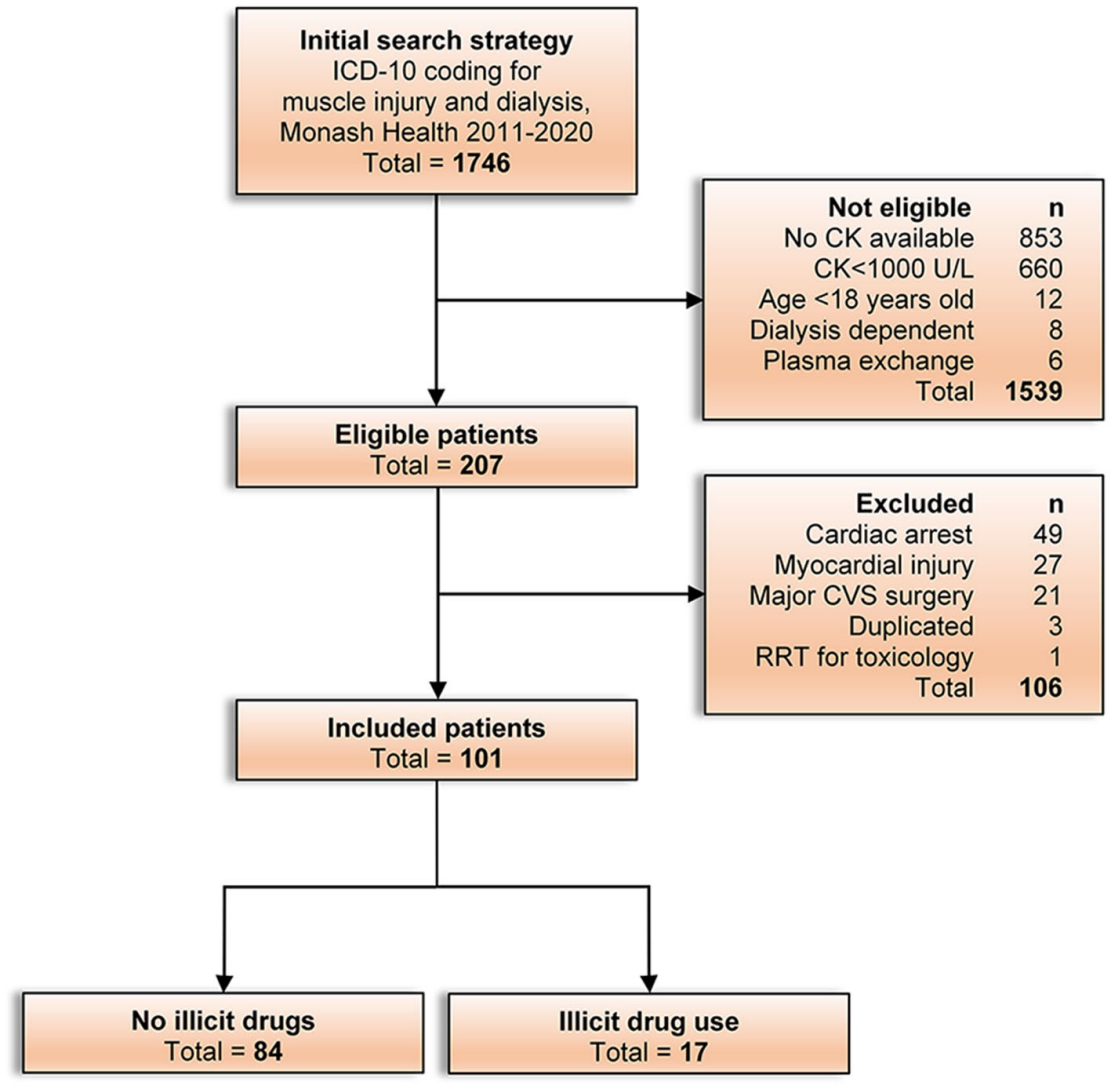
Study flow chart outlining the outcome of the ICD-10 search, assessment of eligibility, number and reasons for exclusions, and the final number of patients included for analysis.

The details of the initial search, number eligible and exclusions are shown in a flow diagram (Figure 1). We included 101 patients in the final analysis. Age was normally distributed and ranged from 18 to 87 years, with a mean of 58 years. Only two patients had dementia and were living in a residential aged care facility. The baseline characteristics of the patients are summarized in Table 1. The median hospital length of stay was 20 days (IQR, 9–28 days). Intensive care admission was required in 93% (94/101) of patients at some point during the admission, and 76% (71/94) required ventilation for a median duration of 128 h (IQR, 64–264 h). A full list of the implicated causes of rhabdomyolysis is presented in the Supplementary Table 1. Illicit drug use was identified in 17% of patients (17/101).

**Table 1:** Baseline characteristics of patients by illicit drug use status.

**Characteristic**	**All patients** ***N* = 101**	**Illicit drugs** ***N* = 17**	**No illicit drugs** ***N* = 84**
Age, mean (SD), years	58.1 (17.8)	44.2 (16.2)	60.9 (16.8)
Male sex, *n* (%)	61 (60.4)	11 (64.7)	50 (59.5)
**Ethnicity**, ***n*** **(%)**			
Caucasian	82 (81.2)	15 (88.2)	67 (79.8)
Asian	13 (12.9)	2 (11.8)	11 (13.1)
Other	6 (5.9)	0 (0)	6 (7.1)
Aged care facility resident, *n* (%)	2 (2.0)	1 (5.9)	1 (1.2)
BMI, median (IQR), kg/m^2^	27.4	26.1	28.0
	(24.2–33.2)	(25.0–30.7)	(23.7–34.0)
Diabetes, *n* (%)	29 (28.7)	2 (11.8)	27 (32.1)
Ischemic heart disease, *n* (%)	27 (26.7)	2 (11.8)	25 (29.8)
Peripheral vascular disease, *n* (%)	10 (9.9)	0 (0)	10 (11.9)
Stroke, *n* (%)	15 (14.9)	1 (5.9)	14 (16.7)
Heart failure, *n* (%)	12 (11.9)	2 (11.8)	10 (11.9)
Cardiovascular disease, *n* (%)^a^	39 (38.6)	3 (17.7)	36 (42.9)
Chronic lung disease, *n* (%) ^b^	18 (17.8)	3 (17.7)	15 (17.9)
Active cancer, *n* (%)	8 (7.9)	1 (5.9)	7 (8.3)
Cirrhosis, *n* (%)	3 (3.0)	0 (0)	3 (3.6)
Dementia, *n* (%)	2 (2.0)	0 (0)	2 (2.4)
Obese (BMI ≥ 30 kg/m^2^), *n* (%)	38 (37.6)	5 (29.4)	33 (39.3)
**CKD stage**, ***n*** **(%)**			
0 or 1, eGFR > 90	62 (61.4)	14 (82.4)	48 (57.1)
2, eGFR 60–89	17 (16.8)	0 (0)	17 (20.2)
3, eGFR 30–59	15 (14.9)	3 (17.7)	12 (14.3)
4 or 5, eGFR < 30	7 (6.9)	0 (0)	7 (8.3)

a *Composite of coronary artery disease, heart failure, stroke, and peripheral vascular disease*.

b *Includes chronic obstructive pulmonary disease, interstitial lung disease, bronchiectasis (excluding simple asthma)*.

*eGFR, estimated glomerular filtration rate by CKD-EPI equation; BMI, body mass index; SD, standard deviation; IQR, interquartile range*.

Patients who used illicit drugs were younger than patients who did not use illicit drugs, with a mean age difference of 16.8 years (95% CI: 7.9–25.6 years, *P* < 0.001). There was also weak evidence that patients who used illicit drugs had less cardiovascular disease (a composite of coronary artery disease, heart failure, stroke and peripheral vascular disease) than those who did not use illicit drugs (18 vs. 43%, *P* = 0.06). There was a similar proportion of patients with CKD (eGFR < 60 ml/min/1.73 m^2^) in those who used illicit drugs compared to those who did not (18 vs. 23%, *P* = 0.76). There were no differences in sex, ethnicity, body mass index (BMI) and diabetes status.

### Pattern of Active Illicit Drug Use

Polysubstance use was common among patients who used illicit drugs. Only 3/17 patients had used a single illicit drug prior to presentation. The categories of illicit drugs were amphetamines or methamphetamines (8/17), benzodiazepines (11/17), opioids including heroin (8/17), cannabinoids (5/17), and cocaine (1/17). There were no cases associated with the use of ecstasy or MDMA identified in this cohort. Although our study period dated back to 2011, 88% (15/17) of these events occurred within the last 5 years (2015 onwards). Most patients had a history of long-term illicit drug use and were known to our addiction medicine unit, and many were forthcoming with information on their drug use.

### Clinical Characteristics

Around 90% of patients had AKI at presentation, and RRT was initiated at a median serum creatinine of 537 μmol/L (Table 2). In 51% of patients, the CK peaked on the day of admission. CK had peaked prior to RRT initiation in 90% of patients and in the remaining patients, the peak occurred within 1 or 2 days after RRT initiation. The distribution of CK was highly skewed, so we performed a logarithmic transformation of CK (log CK) prior to analysis. There was strong evidence that the log CK on admission was higher in patients who used illicit drugs compared to patients who did not (mean difference, log 1.13 U/L, 95% CI: log 0.39–log 2.13 U/L, *P* = 0.005). There was very strong evidence that the log peak CK was higher in patients who used illicit drugs compared to patients who did not (mean difference, log 1.74 U/L, 95% CI: log 0.75–log 2.73 U/L, *P* < 0.001). Exposure to nephrotoxins was common, particularly vancomycin and intravenous contrast. Sepsis and septic shock were also frequently observed in this cohort (Table 2). There was weak evidence for a lower proportion of patients with septic shock in those who used illicit drugs compared to those who did not (29 vs. 55%, *P* = 0.057). Due to the severity of AKI, all patients had renal tract imaging performed, which revealed evidence of obstructive uropathy in two patients that required percutaneous nephrostomy. A kidney biopsy was performed in seven patients, which revealed interstitial nephritis (*n* = 3), small vessel vasculitis (*n* = 3) and thrombotic microangiopathy (*n* = 1).

**Table 2:** Biochemistry, clinical characteristics and dialysis modalities by illicit drug use status.

**Characteristic**	**All patients** ***N* = 101**	**Illicit drugs** ***N* = 17**	**No illicit drugs** ***N* = 84**
Admission SCr, median (IQR), μmol/L	249 (135–401)	272 (178–319)	242 (128–423)
Peak SCr, median (IQR), μmol/L	537 (332–749)	555 (326–849)	530 (346–695)
Admission CK, median (IQR), U/L	2685 (1180–7374)	7320 (4533–40036)	2345 (1154–5860)
Peak CK, median (IQR), U/L	5473 (1795–17051)	18664 (4533–56428)	3892 (1698–10366)
Fluid overload, *n* (%)	47 (46.5)	4 (23.5)	43 (51.2)
Exposure to intravenous contrast, *n* (%)	36 (35.6)	5 (29.4)	31 (36.9)
Exposure to vancomycin, *n* (%)	41 (40.6)	4 (25.5)	37 (44.1)
Exposure to aminoglycosides, *n* (%)	5 (5.0)	2 (11.8)	3 (3.6)
Exposure to any nephrotoxin, *n* (%)^a^	59 (58.4)	8 (47.1)	51 (60.7)
Sepsis syndrome per Sepsis-3, *n* (%)	66 (65.3)	9 (52.9)	57 (65.5)
Sepsis	15 (14.9)	4 (23.5)	11 (13.1)
Septic shock	51 (50.5)	5 (29.4)	46 (54.8)
Hydronephrosis on imaging, *n* (%)	2 (2.0)	0 (0)	2 (2.4)
Intrinsic renal disease, *n* (%)	7 (6.9)^c^	0 (0)	7 (8.3)

a *Composite exposure to vancomycin, aminoglycoside or IV contrast*.

b *Defined per Sepsis-3 criteria reported in Singer et al. (7)*.

c *Biopsy-proven interstitial nephritis (n = 3), vasculitis (n = 3), thrombotic microangiopathy (n = 1)*.

*SCr, serum creatinine; CK, creatine kinase; IQR, interquartile range; CVVHDF, continuous veno-venous hemodiafiltration; SLED, slow low-efficiency dialysis; IHD, intermittent hemodialysis; RRT, renal replacement therapy*.

### Renal Replacement Therapy

**Figure 2:**
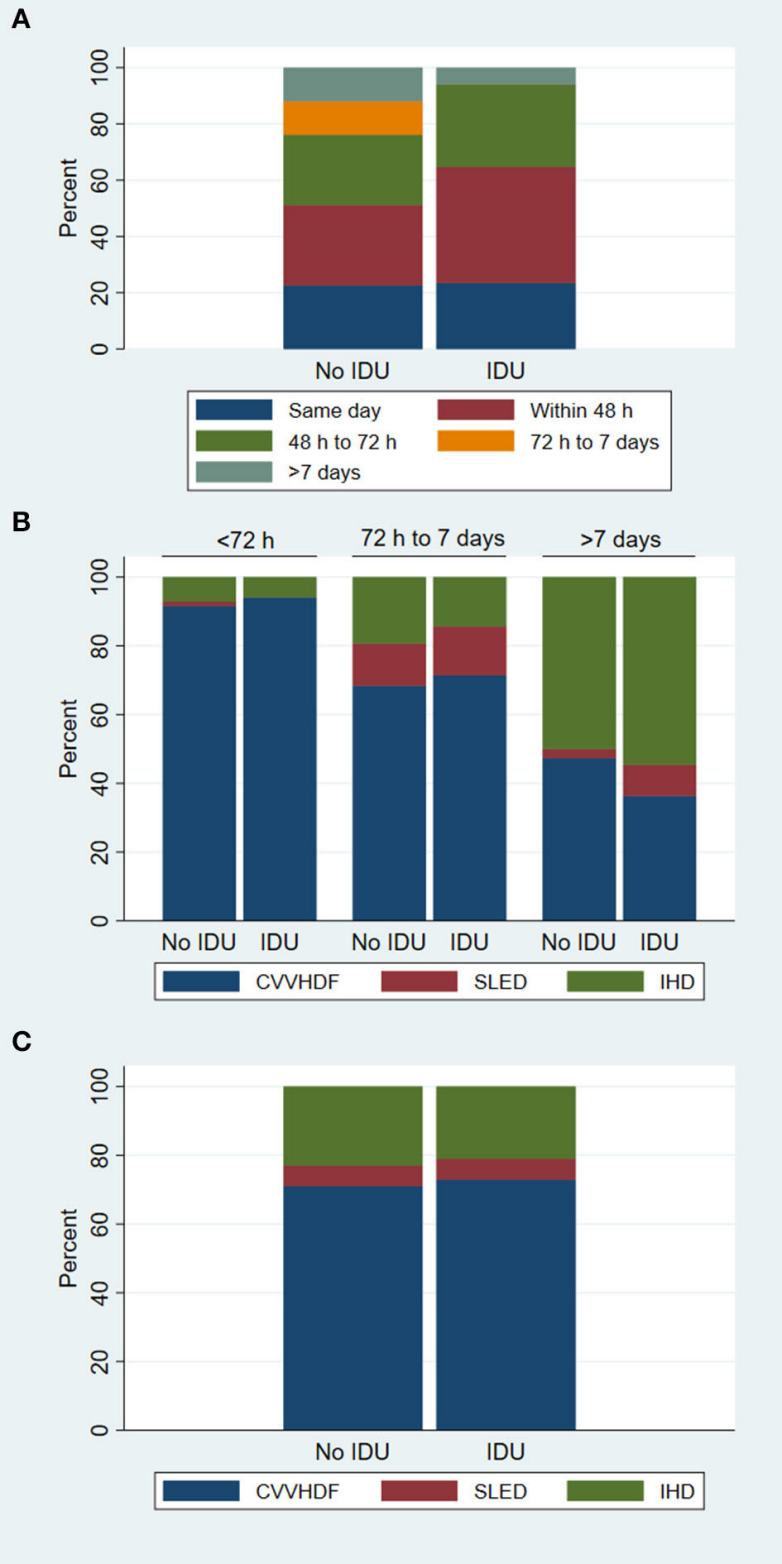
Stacked bar graphs showing the details of renal replacement therapy (RRT) by illicit drug use (IDU) status. **(A)** Shows the timing of RRT initiation after hospital admission. **(B)** Demonstrates the RRT modalities used over time, which included continuous veno-venous hemodiafiltration (CVVHDF), sustained low-efficiency dialysis (SLED) and intermittent hemodialysis (IHD). **(C)** Compares the mean duration of the RRT modalities expressed as a percentage of total RRT time.

A summary of the RRT related information is shown in Figure 2. Despite a small difference in the proportion of patients initiating RRT within 48 h of hospital admission, there was no significant difference in the distribution of peak serum creatinine at RRT initiation between patients with illicit drug use-associated rhabdomyolysis and patients with rhabdomyolysis from other causes (*z* = 0.52, *P* = 0.53). In our cohort, almost all patients initiated RRT with CVVHDF and there was no observable difference in the choice of dialysis modality between patients who used illicit drugs and patients who did not. An equal proportion of patients in each group transitioned to SLED and IHD within a similar timeline. After 1 week, half of patients who remain RRT dependent were transitioned to IHD. Based on these observations, the RRT treatment in both groups appeared similar.

### Survival Models and Outcomes

The total time at risk was 1,273 patient-days. There were 69 events (dialysis independence) and 25 deaths accounting for the competing risk. Two patients were lost to follow up due to transfer to a private facility, and five remained dialysis dependent at the end of the study (90 days), and these seven patients who were censored did not use illicit drugs. There was also no pattern in the cause of rhabdomyolysis in these seven patients (immobilization = 2, infection = 2, medication = 1, seizure = 1, myopathy = 1). Based on the cumulative incidence function, the estimated time to 50% cumulative incidence of RRT independence for the entire cohort was 11 days (95% CI: 8–16 days). Without accounting for the competing risk of death, the overall median duration of RRT was 8.5 days (IQR: 4.5–18.5 days).

In the base model for survival analysis, we included the variables age, log peak CK and sepsis. We chose these variables as they represented differences in baseline variables and clinical characteristics which could confound the association between illicit drug use and the outcome of dialysis independence. In the multivariable analysis, age was not statistically significant (SHR 1.00, 95% CI: 0.99–1.01, *P* = 0.91). When age was dropped from the model, the SHRs for the remaining variables changed by 1% or less. In the final model, we regressed the subdistribution hazard of RRT independence on illicit drug use, log peak CK and sepsis. The estimated SHR for illicit drug use was 1.48 (95% CI: 0.78–2.84, *P* = 0.23), 0.87 (95% CI: 0.76–0.99, *P* = 0.041) for log peak CK, and 0.41 (95% CI: 0.25–0.67, *P* < 0.001) for sepsis.

**Figure 3:**
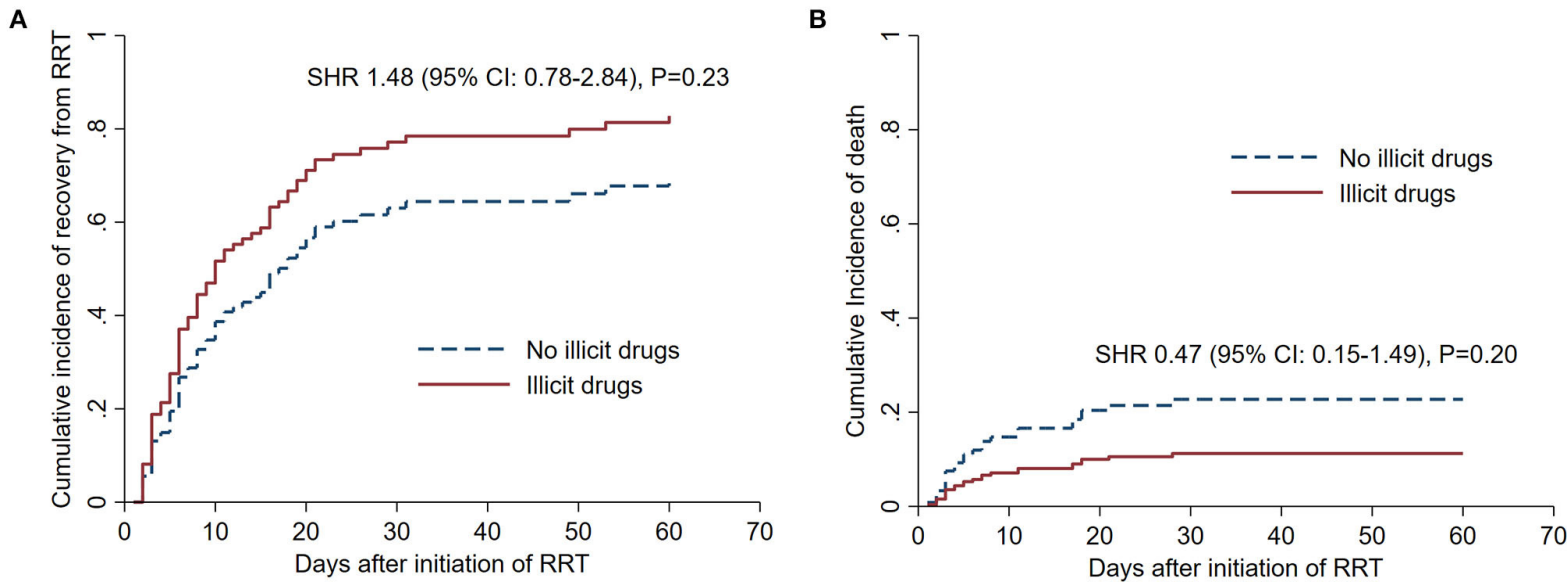
Competing risks survival analysis with subdistribution hazards adjusted for the log transformed peak serum creatine kinase and sepsis, and stratified by illicit drug use status. **(A)** Shows the cumulative incidence curves for independence from renal replacement therapy, and **(B)** shows the cumulative incidence curves for death.

The effect of illicit drug use on the cumulative incidence function of RRT independence and mortality is shown in Figure 3. A comparison between the results of the Fine-Gray competing risks approach and the cause-specific approach is summarized in Table 3. In summary, the small difference in the subdistribution hazard of RRT independence between patients who did and did not use illicit drugs was not statistically significant, suggesting that renal recovery and RRT independence was not worse in patients with illicit drug use-associated rhabdomyolysis.

**Table 3:** Comparison of the competing risks and cause-specific survival models.

**Model/Event**	**Estimates for illicit drug use**	***P* value**
**Competing risks**	**Subdistribution hazard ratio (95% CI)**	
RRT independence	1.48 (0.78–2.84)	0.23
Mortality	0.47 (0.15–1.49)	0.20
**Cause-specific^a^**	**Hazard ratio (95% CI)**	
RRT independence	1.42 (0.75–2.77)	0.29
Mortality	0.49 (0.14–1.72)	0.26

*Models adjusted for sepsis and log peak creatine kinase levels*.

a *Cox regression with censoring for the competing event*.

*RRT, renal replacement therapy*.

### Sensitivity Analysis

We conducted a sensitivity analysis by excluding patients with evidence of intrinsic renal disease on kidney biopsy (*n* = 7) and those with evidence of obstructive uropathy (*n* = 2). The main results did not change. With these patients excluded, the SHR for illicit drug use was 1.41 (95% CI: 0.73–2.73), a change of 4.7% in the estimates.

## Discussion

We previously found an association between illicit drug use and a higher risk of AKI and RRT in patients with rhabdomyolysis (9), which we postulated may be due to the direct nephrotoxicity of illicit drugs in addition to the well-known mechanisms of renal injury involving myoglobinuria. We have now extended our investigation to determine if illicit drug use affects the renal prognosis among those with severe AKI needing RRT, using the duration of RRT as a marker for renal recovery. Our current study has demonstrated that rhabdomyolysis in patients who used illicit drugs was not associated with a worse renal prognosis for recovery from RRT, compared to rhabdomyolysis from all other causes.

The majority of studies in patients with rhabdomyolysis focused on risk factors or prediction of AKI and RRT (3, 10, 11), but the RRT duration is not always reported. Even a Cochrane review on continuous RRT for rhabdomyolysis did not include RRT duration as an outcome of interest (12). We estimated from the competing risks analysis a duration of RRT of 11 days at 50% cumulative incidence, but a median duration of 8.5 days when disregarding the competing risk of death. Contrary to our original hypothesis that illicit drug use may be associated with a worse renal outcome, the cumulative incidence function suggested the prognosis may be in fact better in these patients. In our study, it was rare for survivors to remain dialysis dependent. This finding is consistent with most studies. A recent French study of 103 patients needing RRT (of 315 patients with AKI) for rhabdomyolysis-associated AKI reported only one patient needing dialysis at 3 months. However, the French investigators showed that 30% had significant residual impairment. Progression to advanced CKD (eGFR < 30 ml/min/1.73 m^2^) was also relatively common (11). A smaller study by Rodriguez et al. reported that RRT was maintained for 8.3 (range, 2–21) days in 12 patients who needed RRT (of 73 patients with AKI), and no patient remained dialysis-dependent at discharge (3). In patients with heroin-related rhabdomyolysis and AKI, 8/27 patients required RRT for an average of 14 days (range 3–26 days) and all became dialysis-free (13).

There is a possibility that RRT itself alters the outcomes by removing myoglobin from the circulation. The molecular weight of myoglobin is approximately 17 kDa. With the Prismaflex ST100 set (surface area, 1 m^2^), the reported *in vitro* sieving coefficient for myoglobin is 58% (blood flow 100 mL/min, ultrafiltration 20 mL/min). In clinical practice, this is usually much lower and tends to decline over the life of the filter. CVVH using a similar AN69 (surface area, 0.9 m^2^) membrane (blood flow 150 mL/min, ultrafiltration 35 mL/min) was associated with an average myoglobin sieving coefficient of 60% initially which declined to 40% after 9 h, associated with an initial myoglobin clearance of 22 mL/min and dropping to 14 mL/min (14). Although standard hemodialysis membranes do not allow myoglobin passage, the reported myoglobin clearance using a high-flux dialyzer (Ultraflux AV1000S, surface area 1.8 m^2^, 30 kDa cut-off) averaged 90 mL/min (blood flow 200 mL/min) (15). Therefore, it is possible that RRT and IHD may promote myoglobin clearance by extracorporeal means, but we cannot know if this is clinically relevant. To our knowledge, there are no studies showing the benefits of early or aggressive RRT on renal recovery or mortality in patients with rhabdomyolysis-associated AKI, compared to routine RRT used for standard indications of AKI (12). In our study, we demonstrated that dialysis modalities and timing of RRT initiation were balanced in the groups compared, thus minimizing treatment-related bias. However, whether the overall duration of RRT is affected by the choice of RRT filters and modalities cannot be answered in this study.

In our survival models, we noted a statistically significant effect of sepsis and peak CK on the SHR of RRT independence. However, these covariates were used to adjust for confounding of the primary effect of illicit drug use on RRT outcomes. Sepsis as a confounder is not surprising. Sepsis is commonly observed in patients who present with rhabdomyolysis, and in one study 9.4% patients with rhabdomyolysis died of septic shock (3). The confounding effect of peak CK levels is novel from our perspective. We have previously shown that compared to other causes, illicit drug use-associated rhabdomyolysis is associated with higher peak CK levels in hospitalized patients but this association itself is also confounded by age (9). We have not come across any studies which have determined if peak serum CK levels influences RRT duration in patients with rhabdomyolysis. Thus, we suggest that sepsis and log peak CK are variables which should be considered in future studies where prediction of RRT outcomes are of interest in patients with rhabdomyolysis.

In our study, the mortality rate was 25%. This is relatively low compared to other studies. We suspect this is due to our exclusion criteria. To reduce confounding, we attempted to create a more restricted cohort by excluding patients with rhabdomyolysis following cardiac arrest and patients who had emergency major cardiovascular surgery, such as abdominal aneurysm repair and coronary artery bypass grafting. Such patients can have profound sustained shock and a high mortality rate. The RRT outcomes in such patients depend on the adequacy and success of resuscitation, revascularization, and restoration of perfusion (cardiopulmonary bypass, intra-aortic balloon pump, aortic cross-clamping), which introduces complex confounding and heterogeneity. In principle, the AKI is these patients is mostly due to severe ischemia rather than rhabdomyolysis. In studies without our strict exclusion criteria, mortality as high as 50% was reported (16).

To our knowledge, this study has one of the largest cohorts of RRT-requiring rhabdomyolysis-associated AKI patients reported in the literature. Even a recent French multi-center study of eight ICUs could only include 103 patients with severe rhabdomyolysis and AKI needing RRT (11). Another strength is the use of survival analysis which accounted for the high mortality in this population. Without accounting for the competing risk of death, studies may underestimate the required duration of RRT. For example, one previous study reported the mean duration of continuous RRT required as 5.5 days (SD, 4.3 days), but there was a 50% mortality in this group and most of the deaths occurred <3 days after initiation of RRT (16). Even our own estimates of the median duration of RRT was shorter than the equivalent estimate from the cumulative function allowing for competing risk.

The main limitations of the study were its observational design, leading to susceptibility to bias and inability to infer causality. Outside of a trial setting, the decision to initiate and cease RRT is subject to variation between intensivists and between nephrologists. Without controlling for the modality and intensity of RRT, there may be a treatment bias affecting the outcome. Due to the small number of patients who used illicit drugs, a type 2 error may exist, and larger studies may find an association between illicit drug use and different outcomes in patients with rhabdomyolysis. However, our primary interest for conducting the study was to determine if patients who used illicit drugs had a worse renal prognosis and therefore a lower hazard of RRT independence. In that respect, we considered a HR of 0.70 as a clinically significant difference. In terms of the inference for the population, we point out that the 95% confidence interval for the cause-specific HR did not include 0.70. We believe that it is reasonable to infer that patients who used illicit drugs are unlikely to have a clinically significant lower hazard for achieving RRT independence following rhabdomyolysis and severe AKI, compared to rhabdomyolysis from other causes. In terms of generalizability, our study did not include patients with cardiac arrest or patients who proceeded directly to emergency cardiovascular surgery. Therefore, any inferences from our study does not apply when such patients are included. There were also very few patients who received isolated IHD, so a similar caution applies to settings where rhabdomyolysis patients are initiated and maintained entirely on IHD. Certain etiologies of rhabdomyolysis were under-represented, such as exertional injuries and inflammatory muscle diseases.

In conclusion, we could not reject the null hypothesis that patients presenting with rhabdomyolysis associated with illicit drug use, who needed RRT, have the same renal prognosis for RRT independence as patients who did not use illicit drugs. Thus, although patients who used illicit drugs have a previously identified higher risk of needing RRT, we did not find evidence that their short-term renal prognosis was worse than patients with rhabdomyolysis from other causes. However, further research would be useful to determine if patients who used illicit drugs and developed rhabdomyolysis-associated AKI requiring RRT have a higher risk of CKD or CKD progression in the longer term, when compared to rhabdomyolysis from other causes.

## Data Availability Statement

The datasets presented in this article are not readily available because its availability is subject to approval by the institutional ethics committee or research directorate. Requests to access the datasets should be directed to Andy K. H. Lim, andy.lim@monash.edu.

## Ethics Statement

The studies involving human participants were reviewed and approved by Monash Health Human Research Ethics Committee. Written informed consent for participation was not required for this study in accordance with the national legislation and the institutional requirements.

## Author Contributions

AL conceived and designed the study, performed the statistical analysis, and drafted the manuscript. MA, JP, WL, and CB collected the data. All authors contributed to the critical review, revision of the manuscript, and read and approved the final submitted version of the manuscript.

## Conflict of Interest

The authors declare that the research was conducted in the absence of any commercial or financial relationships that could be construed as a potential conflict of interest.
