# Supplementary material for: The Association Between Illicit Drug Use and the Duration of Renal Replacement Therapy in Patients With Acute Kidney Injury From Severe Rhabdomyolysis
Source: Front Med (Lausanne). 2020 Nov 9;7:588114. doi: 10.3389/fmed.2020.588114 (PMC7680872; doi:10.3389/fmed.2020.588114)
Supplement: Supplementary file 1 [file Table_1.pdf]

Table S1. Implicated causes of rhabdomyolysis (n = 101)

Category [a]	Number	Percent of cohort
Trauma	22	21.8
Operative	13	12.9
Acute ischemia or muscle bleeding	14	13.9
Pressure injury	38	37.6
Exertional	0	0
Seizure	5	4.6
Infection-related	33	32.7
Drugs and toxins	30	29.7
Inflammatory myopathies and myositis [b]	2	2.0
Severe electrolyte or metabolic disorder [c]	7	6.9
Familial or inherited diseases [d]	1	1.0
Thermal extremes and dysregulation [e]	3	3.0
Uncertain cause	2	2.0

[a] Categories are not mutually exclusive due to multiple mechanisms in some patients.

[b] Polymyositis, dermatomyositis, inclusion body myositis and autoimmune disease with myositis.

[c] Serum sodium <120 mmol/L, potassium <2 mmol/L, phosphate <0.4 mmol/L and diabetic ketoacidosis.

[d] Metabolic myopathies, inborn errors of metabolism, mitochondrial disease.

[e] Heat stroke, malignant hyperthermia, severe hypothermia and near drowning.
